# Qualitative research with patients and caregivers of patients with PIK3CA related overgrowth spectrum: content validity of clinical outcome assessments

**DOI:** 10.1186/s41687-022-00481-8

**Published:** 2022-07-13

**Authors:** Kimberly Raymond, Susan Vallow, Cory Saucier, Kristi Jackson, Michelle K. White, Andrew Lovley, Denise D’Alessio

**Affiliations:** 1 QualityMetric, Johnston, RI USA; 2 Novartis Pharmaceuticals, East Hanover, NJ USA

**Keywords:** PIK3CA related overgrowth spectrum, Patient experience, Content validity, Clinical outcomes assessment, Cognitive debriefing, Health-related quality of life

## Abstract

**Background:**

PIK3CA-Related Overgrowth Spectrum (PROS) are rare syndromes caused by a mutation in the PIK3CA gene, including fibroadipose hyperplasia or overgrowth; congenital lipomatous overgrowth, vascular malformations, epidermal nevi, scoliosis/skeletal and spinal (CLOVES); megalencephaly-capillary malformation (MCAP or M-CM); fibro-adipose vascular anomaly (FAVA); Klippel-Trenaunay syndrome (KT; also known as, Klippel-Trenaunay-Weber syndrome); capillary, lymphatic, and venous malformations (CLVM); and lymphatic malformation (LM). Characterized by malformations and tissue overgrowth, PROS manifests at birth or in early childhood. Pain and functional limitations associated with these conditions may greatly impact the health-related quality of life (HRQoL) of persons with PROS including physical functioning, work/school, social functioning, and emotional well-being.

**Results:**

Selected clinical outcome assessments (COAs), identified during a literature review, were tested with adults with PROS, and children with PROS and their caregivers to determine comprehensibility, relevance, and appropriateness for measuring symptom severity and HRQoL. Tested were the Patient Global Impression of Symptom Severity (PGI-S), Brief Pain Inventory (BPI), Wong-Baker FACES, Patient-Reported Outcomes Measurement Information System (PROMIS) Profile, PROMIS Pediatric Short Form Sleep Disturbance, and PROMIS Dyspnea Severity. Qualitative interviews tested the self-report adult, self-report pediatric, and observer-report COAs with adults with PROS, and children with PROS and their caregivers.

Ten adults (≥ 18 years old) with PROS, and 20 children (6–17 years old) with PROS and their caregivers, participated. All reported positive feedback on item relevance. Adults and children over the age of 12 comprehended and responded to self-reported items. Secondary objectives examined the age children could self-report their conditions using pediatric versions and assessed available observer-report versions of the COAs with caregivers. Some participants under the age of 12 had trouble understanding some terminology. Further, adults and children with cognitive impairment associated with MCAP/M-CM sometimes had difficulty with self-report. Caregivers were able to report their child’s symptoms and impacts using observer-report COAs. Participant feedback prompted further consideration of the measurement of pain in this population, including variability of pain over time, location of pain, and type.

**Conclusions:**

This study provided valuable information from patients about PROS, supporting the content validity of the COAs, with recommended revisions. COAs are easily understood by persons with PROS and caregivers and are appropriate for measuring symptoms and disease-related impacts across diverse PROS syndromes in clinical trials.

## Introduction

PIK3CA-related overgrowth spectrum (PROS) is an umbrella term describing a heterogeneous group of rare syndromes caused by a mutation in the PIK3CA gene and characterized by malformations and overgrowth of adipose tissue, muscle, skin, bone, blood or lymph vessels, or neural tissues [1–4]. Syndromes included under PROS are fibroadipose hyperplasia or overgrowth; congenital lipomatous overgrowth, vascular malformations, epidermal nevi, scoliosis/skeletal and spinal (CLOVES); megalencephaly-capillary malformation (MCAP or M-CM); fibro-adipose vascular anomaly (FAVA); Klippel-Trenaunay syndrome (KT; also known as Klippel-Trenaunay-Weber syndrome); capillary, lymphatic, and venous malformations (CLVM); and lymphatic malformation (LM) [5, 6]. Irrespective of syndrome, PROS may cause extreme pain, impacting the health-related quality of life (HRQoL), of persons with PROS, including social and emotional well-being, the ability to function physically and fulfill household, work, and school-related roles.

A separate literature review was conducted prior to the current study to identify a comprehensive list of important concepts which capture the expected heterogeneity of symptoms across PROS syndromes [7]. These concepts were then matched to existing clinical outcome assessments (COAs), following regulatory guidance documents for medical product development [8–10]. In rare diseases such as PROS, it is common to investigate whether existing tools can adequately measure concepts, rather than adapting or developing new measures [11]. This literature review revealed that no single COA existed that covered the range and diversity of symptoms in this condition. Further, there was no consensus in the literature as to which COAs should be used to capture this information across PROS syndromes. The current study was, therefore, designed to test whether a selected group of COAs, which included concepts identified in the literature as relevant to PROS, were adequate for use in this population.

### Objectives

Content validity of selected COAs measuring pain and other symptoms and impacts commonly experienced by persons with PROS were tested in preparation for a clinical trial in PROS. These COAs were tested using cognitive debriefing (CD) techniques in individual qualitative interviews. While the selected COAs were widely used and validated for use in other health conditions, they had not been tested for use in this population. This study also aimed to establish an age threshold for self-report among children with PROS (6–17 years old), to determine when the self-report or observer-report (measurements completed by a parent, caregiver, or someone who observes the person with PROS daily) versions of the COAs would be most appropriate.

## Methods

### Study design

This qualitative, non-interventional, cross-sectional study was designed in accordance with regulatory and industry best practices for establishing content validity of COAs [8, 10, 12]. CD interviews were conducted in early 2020, via telephone or webcam, with adults with PROS, and children with PROS and their caregivers. Interviews were designed to determine whether concepts covered in the COAs were relevant, items were easily understood by persons with PROS, and the COAs collectively covered the concepts relevant to persons with PROS to measure symptoms and impacts in this population. Ethics approval for the study was granted by the New England Independent Review Board.

### Study participants

A sample size of 30 participants was determined based on commonly used standards regarding the number of interviews needed to test understandability of items and relevance of concepts [8, 12, 13]. Interviews were conducted with two groups of participants: adults ≥ 18 years old (n = 10), and children 6–17 years old, with their caregiver (n = 20). All children and their caregivers were invited to participate in the interviews. A purposive sampling approach was used to construct a sample whereby COAs could be tested across PROS syndromes, and to achieve variation in characteristics, such as geographic residence, sex, race, and age. Children from five different age groups (6–7 years, 8–9 years, 10–12 years, 13–14 years, and 15–17 years) were purposefully recruited and interviewed to determine the appropriate age for self-report. An additional quota wherein 40–60% of participants had a diagnosis of either CLOVES or KT was established to align with the percentage of these diagnoses in the literature and the expected proportion in the target clinical trial.

Participants were recruited from across the United States (US) through partnerships with five patient advocacy groups, a vendor specializing in healthcare recruitment, and this vendor’s social media and physician partners. Eligibility for participation required that individuals live in the US, be fluent in English, and have a doctor's diagnosis of PROS; specifically, CLOVES, KT, CLVM, MCAP or M-CM, FAVA, or LM. Adult participants were required to be ≥ 18 years old, and child participants were required to be between 6–17 years old and have a caregiver also willing to participate. All provided written informed consent or assent, and caregivers provided signed permission for their minor child to participate.

### Interview procedure

Individual interviews (75 min, by webcam or phone) were conducted by experienced qualitative researchers using a semi-structured interview guide. Warm-up questions established rapport and gathered basic information about participants’ experiences with PROS. Participants completed a battery of COAs using a think-aloud CD approach [14]. They were asked to verbalize their thoughts while responding to the items of each COA, articulating the instructions, items, and response choices [12, 13]. Next, participants were asked structured questions about each COA item to assess relevance, comprehensiveness, and comprehensibility. Participants were asked to comment on whether items were relevant to their condition and were understandable, and whether any concepts were missing that would be important to capture to fully understand the symptoms and impacts of their condition. Participants were also asked whether they understood the items (e.g., terms) and further probing also determined whether they correctly understood the meaning of the items as intended (Table 1). Participants also commented on each COA’s instructions, recall period, and response choices, and noted any aspects they found confusing. Additional probes explored their experiences of pain to gather information to determine an appropriate approach for measuring pain in this condition. These probes asked for a description of the pain, location, variability, and impacts of pain on daily activities.

**Table 1:** Sample interview guide questions

Instrument component	Sample interview guide question
Relevance	Do these questions apply to your experiences with PROS? Do any NOT apply? Which ones did you relate to the MOST? *
Comprehension	How easy or difficult was it for you to answer this question? **
Comprehensiveness	Were there important parts of your experience with PROS that were not covered by these questions? ***

*Question asked for each instrument; ** Question asked for each item of the instrument; ***Question asked about the full battery of COAs

Three sets of COAs were tested during this study: one set was completed by adults ≥ 18 years old (adult self-report), another by children 6–17 years old (pediatric self-report), and a third by caregivers of those children 6–17 years old (observer-report). Caregiver participation was minimal when children were able to self-report. In instances where children were unable to self-report, the interview, either partially or in full, was conducted with the caregiver (n = 10) to test observer-report versions of the instruments. The COAs tested in this study were the Patient Global Impression of Symptom Severity (PGI-S), Brief Pain Inventory (BPI), Wong-Baker FACES, Patient-Reported Outcomes Measurement Information System (PROMIS) Profile, PROMIS Pediatric Short Form Sleep Disturbance, and PROMIS Dyspnea Severity [15–21]. Specific information pertaining to the COAs tested, including versions and the respective patient population tested, is detailed in Table 2.

**Table 2:** List of COAs included in cognitive debriefing interviews

Concept of interest	COA by report		
	Adult self-report	Pediatric self-report	Observer-report
Global impression of symptom severity	Single item on the overall severity of PROS^a^		
Pain intensity	Brief pain inventory, items 2-6^d^	Brief Pain Inventory, items 2-6^d^	–
		Wong-Baker FACES	
Physical function	PROMIS-29 + 2 Profile v2.1	PROMIS Pediatric-25 Profile v2.0	PROMIS-Parent-Proxy-25 Profile v2.0
Fatigue			
Ability to participate in social roles and activities/Peer relationships^b^			
Pain interference			
Pain intensity			
Anxiety			
Depression			
Sleep disturbance^c^		PROMIS Pediatric Short Form v1.0 – Sleep Disturbance 4a	PROMIS Parent Proxy Short Form v1.0 – Sleep Disturbance 4a
Dyspnea	PROMIS Dyspnea Severity, three items from item bank		–

^a^While the overall content of these items were identical across the adult, child, and observer-reported measures, minor changes to item wording were made to make it relevant for the intended group (e.g., modifying ‘how would you rate your overall symptoms’ to ‘how would you rate your child’s overall symptoms)

^b^ ‘Ability to participate in social roles and activities’ was assessed for adults, while ‘peer relationships’ was assessed for children/observer-report

^c^Sleep disturbance was assessed in the PROMIS-29 + 2 Profile v2.1 using the PROMIS Sleep Disturbance Short Form 6a. Sleep disturbance was not assessed on the PROMIS Pediatric or Parent Proxy Profile, and thus a separate short form assessment was needed

^d^Brief Pain Inventory Items 2–6 were as follows: (2) On the diagram, shade in the areas where you feel pain. Put an X on the area that hurts the most; (3) Please rate your pain by circling the one number that best describes your pain at its worst in the last 24 h; (4) Please rate your pain by circling the one number that best describes your pain at its least in the last 24 h; (5) Please rate your pain by circling the one number that best describes your pain on the average; and (6) Please rate your pain by circling the one number that tells how much pain you have right now

Child/caregiver interviews were structured similarly to the adult interviews, except that warm-up questions had an additional goal of assessing the child’s ability to understand and respond to self-reported items. If it became evident that the child was unable to comprehend or respond to the items, the interviewer switched to testing the observer-report versions with the caregiver. This strategy determined the relevance, comprehensiveness, and comprehensibility of observer-report COAs, and whether caregivers believed they could accurately report observed symptoms and impacts of the child’s condition.

### Data analysis

Interviews were audio-recorded and transcribed verbatim. Final transcripts were pseudonymized to ensure identifying information was excluded. Preliminary coding was conducted after each interview, using Microsoft Excel, to capture findings in a standardized format from field notes collected during the interview. Once transcripts were completed and quality checked, formal coding was conducted by two experienced qualitative researchers using narrative data from the interviews. Formal coding included a comparison of transcripts to preliminary coding files, and extraction of further participant feedback on all COA components including instructions, recall period, item response options, and the relevance and comprehension of each item. All responses reported by participants were evaluated by the research team and assigned codes to capture the type of feedback and whether remarks were spontaneous or prompted after questioning by the interviewer. Comments reported by participants pertaining to item comprehension and the ability to respond accurately to items were noted. Discrepancies between coders were resolved by the research team to ensure agreement in coding. All coding was then reviewed and confirmed against transcripts by a second member of the study team and reviewed and finalized by the study primary investigator (PI). Participants’ descriptions of pain and most bothersome symptoms were coded separately using grounded theory methods and NVivo qualitative software, version 12.0 [22]. Given the expected heterogeneity of the sample, all data were analyzed collectively and considered independently for each PROS syndrome.

## Results

### Participant characteristics

Adult participants were predominantly female (n = 7; 70%) and Caucasian/White (n = 8; 80%) and ranged from 25–72 years of age (mean = 38.9; SD 14.46). Adult participants were interviewed across diverse PROS syndromes, with 60% (n = 6), as planned, reporting a diagnosis of either KT or CLOVES. A single participant was diagnosed with both KT and CLOVES. Individuals with MCAP/M-CM (n = 2; 20%) and FAVA (n = 1; 10%) were also interviewed. Child participants were also predominantly female (n = 14; 70%) and Caucasian/White (n = 17; 85%) and ranged from 6–17 years of age (mean = 11.8; SD 3.81). Child participants were diagnosed with KT (n = 6; 30%), CLOVES (n = 4; 20%), MCAP/M-CM (n = 3; 15%), FAVA (n = 5; 25%), and LM (n = 2; 10%). Specific sample characteristics for adult and child participants are reported in Tables 3 and 4, respectively.

**Table 3:** Adult (> 18 years) Participant Demographics and Characteristics

	Total population (*N* = 10)
*Age, n (%)*	
18–30 31 +	3 (30.0) 7 (70.0)
Female, n (%)	7 (70.0)
*Race, n (%)*	
Non-Hispanic/White Hispanic/Latino Did not wish to answer	8 (80.0) 1 (10.0) 1 (10.0)
*Geographical Location, n (%)*	
US—North US—South US—East US—West	2 (20.0) 3 (30.0) 3 (30.0) 2 (20.0)
*Syndrome, n (%)*	
KT CLOVES KT & CLOVES MCAP/M-CM FAVA LM	5 (50.0) 1 (10.0) 1 (10.0) 2 (20.0) 1 (10.0) 0 (0.0)

**Table 4:** Child (6–17 years) Participant Demographics and Characteristics

	Total population (*N* = 20)
*Age, n (%)*	
6–7 8–9 10–12 13–14 15–17	4 (20.0) 2 (10.0) 4 (20.0) 5 (25.0) 5 (25.0)
Female, n (%)	14 (70.0)
*Race, n (%)*	
Non-Hispanic/White Hispanic/Latino Black or African American	17 (85.0) 2 (10.0) 1 (5.0)
*Geographical location, n (%)*	
US—North US—South US—East US—West	2 (10.0) 2 (10.0) 7 (35.0) 9 (45.0)
*Syndrome, n (%)*	
KT CLOVES MCAP/M-CM FAVA LM	6 (30.0) 4 (20.0) 3 (15.0) 5 (25.0) 2 (10.0)

### Cognitive debriefing results

Adult participants stated that, overall, the symptoms and impacts measured were appropriate and relevant to their experiences with PROS, and that items were easy to understand and comprehensively captured their experiences. No problems were consistently reported by participants regarding COA instructions, recall period, response options, or comprehensibility of items. Some adult participants had difficulty answering specific items of the BPI and PROMIS Profile related to average pain due to the variability of pain they experienced throughout the day. Child participants stated that symptoms and impacts measured by the pediatric versions of the COAs tested were appropriate, relevant to their condition, and comprehensively captured their experiences with PROS. Difficulties answering questions about average pain reported by adult participants were echoed in the child interviews. Specific feedback for each COA tested is reported below. Sample sizes and percentages reflect the number of individuals who provided feedback on a specific follow-up question during the interview. Recommended changes to tested COAs based on participant feedback are included in Table 5.

**Table 5:** Recommended changes of tested COAs for use in a PROS population

Study finding	Recommended change to COA for use in PROS
The ability to comprehend and self-report differed based on age and syndrome of the participant. Participants under 12 years of age were not able to consistently self-report; adult and child participants’ ability to self-report may need to be determined on an individualized basis in relation to their syndrome	Pediatric versions of COAs included in this battery are best used for individuals ages 12 -17, while observer-report versions are most appropriate for those below the age of 12
	Individuals suffering from cognitive impairment might not be able to self-report, in which case caregiver observer-report would be most appropriate
The variability of patients’ symptom experience within and across days suggested the need for frequent assessment of symptoms. Also, due to the variability of experiences of pain and other symptoms throughout the day, many participants were struggling with questions that asked them to report averages	The BPI item measuring “average” pain (BPI-SF item 5) may not be appropriate in this population, due to participants’ difficulty selecting a response on this item. The BPI item measuring “worst pain” (BPI-SF Item 3) may be most appropriate to assess pain intensity, as participants reported this item as relevant, understandable, and easy to select a response
	The PGI-S was reported by both adults and children as difficult to respond to due to variation of symptoms, both within and across days, and the sheer number of symptoms experienced as part of this condition. Frequent assessment may be useful to capture both good and bad days due to fluctuating symptoms. For example, Ecological Momentary Assessments (EMAs) or Experience Sampling Methods (ESMs) could be appropriate, if tested for use in this population to gather daily changes in symptoms. Additionally, a more specific recall period, such as “past 24-h,” would standardize the period being measured for each participant
Participants acknowledged different aspects of their pain, both in relation to pain type and pain location. Each of these aspects may vary within a particular day and across days	Aspects of pain that participants thought important to describe their pain at a given point in time included pain type, location, intensity, and severity. The battery of COAs tested did not include an item to measure pain type. Future use of these measures in this population should consider whether an item related to pain type is important for its purpose
	Given that pain in PROS often occurs in multiple areas of the body, an option to report more than one location should be captured. Patients also identified pain type as an important concept to measure. Including the BPI pain type item may therefore provide a more comprehensive measure of pain in PROS patients
While children understood the Wong Baker FACES scale, nearly half reported difficulty selecting a response as they struggled to identify with the face associated with the numerical rating scale	While the Wong-Baker FACES scale is validated for use in ages 3 and up, the BPI may be a more appropriate tool for capturing the nature of pain experienced by children with PROS, aged 12 and over

#### Patient global impression of severity

Adults (8/10, 80%) reported the PGI-S item as relevant. This item asked them to rate the overall severity of symptoms they experienced “today.” Most (7/10, 70%) indicated no difficulty responding to this item. Three (30%) stated the item may be too vague to capture mutable characteristics of their symptoms, though they were able to consider symptoms they experienced throughout the day to select an accurate answer. Out of 17 children, 14 (82%) had no difficulty comprehending this item, which has not been modified from the adult version for a pediatric population. Three children (6–7 years old) had trouble reading and understanding the item as written. Both adult and child participants expressed concern with using this single state scale to rate overall severity, when considering the multiple symptoms of PROS, which varied in severity throughout the day. However, this was not reported consistently by participants and did not interfere with the ability to respond to the item.

#### Wong baker FACES pain rating scale

This item was only debriefed with child participants (6–17 years old). Twelve out of 15 (80%) children reported this item as relevant. Twelve out of 17 (71%) reported no difficulty comprehending the item, though 3 (18%), 6–8 years old, had difficulty reading and understanding the item. The Wong Baker FACES is validated for use in ages 3 and over; however, nearly half of the older children (> 12 years old) found the faces to be juvenile and not matching how they felt and experienced pain. Those over 12 years old preferred the BPI items to capture pain intensity.

#### Brief pain inventory items 2–6

**Fig. 1:**
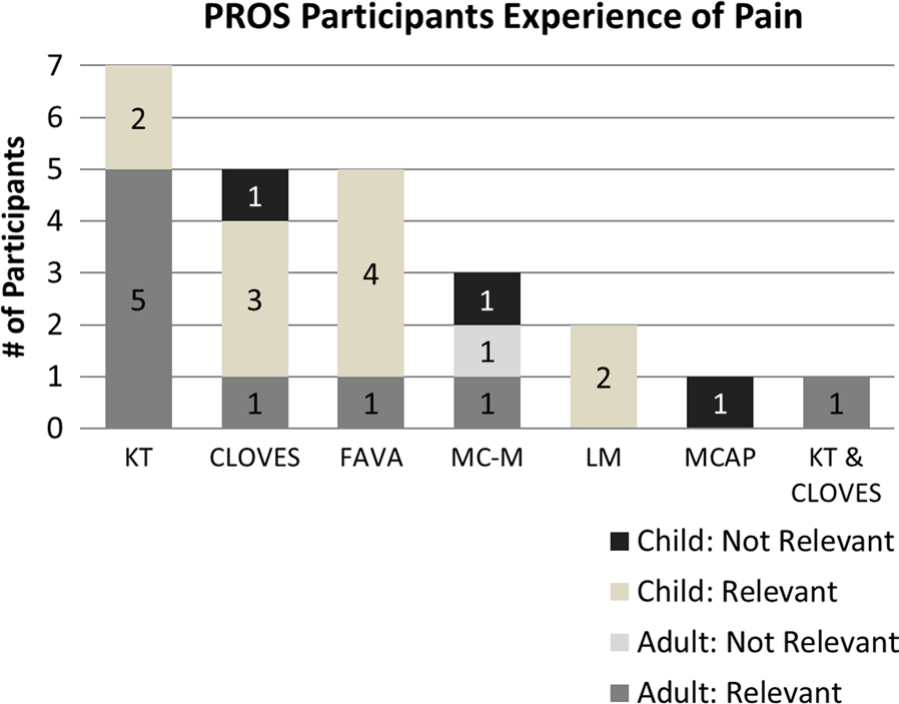
Adult and child participants’ experiences of pain

Adult participants (9/10, 90%) reported all tested items of the BPI (items 2–6) as relevant and easy to comprehend. Six out of 10 (60%) had trouble with item 5, which asked them to rate their average level of pain in the past 24 h, due to the variation in pain they experienced throughout the day. Participants generally preferred items that asked about their pain and symptoms “right now,” as it was easier for them to think about how they were feeling in the moment. Participants also preferred to report on their “worst pain” rather than “average pain,” as they were concerned that reporting average pain would fail to reflect the true severity of their symptoms. Out of 15 child participants, 14 (93%) reported the items of the BPI as relevant. Ten (67%) reported no difficulty comprehending or responding to the items, while 3 (20%), 7–11 years old, struggled with the meaning of the term “average,” and 2 (13%) struggled to choose an answer related to average pain. Many adult and child participants preferred an option to report multiple locations for pain using the BPI diagram, because their pain was not always limited to a single location. They also said that some aspects of pain, such as pain type (e.g., shooting pain, sharp pain, dull pain), were not captured in the current battery but would provide a more comprehensive image of their pain. Follow-up probes were asked to better capture participants’ experiences of pain, which revealed pain as the most salient symptom, across syndromes, due to its impactful, continuous presence in their day to day lives (See Table 6 and Fig. 1). A total of 8 participants (27%), consisting of 2 adult participants, 2 caregivers, and 4 child participants were asked which symptom would be the most important to improve with treatment. Five of the 8 (63%) selected pain as the most important symptom to treat.

**Table 6:** Relevance and experience of pain for adult and child participants by syndrome

Syndrome	Number (n) of interviews conducted by syndrome	Relevance of pain reported by syndrome n (%)	Representative quote [age in years]
*KT*			Um, on a day-to-day basis it—I, I would more describe it as an ache. Um, and if you think, the best way I've seen in literature and things is like that old man's weather ache, just that it's just been a constant ache that—and it—and it just makes you a little bit tired. [Adult, Age 38] Um, sometimes I get sharp pains, um, up through my leg and sometimes it's just like an achy tiredness throughout my leg. [Child, Age 14]
Adult	5	5 (100%)	
Child	6	6 (100%)	
*CLOVES*			It's, it's like a throbbing pain… it happens any time I just put, you know, any sort of pressure on, on my leg. So, if I'm walking, like, a far distance or if I'm running, erm, that pain… And the pain doesn't start right away. The pain'll start, like, later that evening, so maybe, like, three hours or four hours after; and then it'll last probably, like, four hours; [chuckles] and then it goes away! [Adult, Age 37] It comes with a lot, like, of chronic pain for a lot of people, erm, so I usually am in pain somehow! [Child, Age 17]
Adult	1	1 (100%)	
Child	4	3 (75%)	
*KT & CLOVES*			Erm, so my hands it's like a throbbing, like a pulsing kind of feeling, erm, like kind of like your veins are exploding… and then my hip is, is like a buzzing, it's like a, almost like, how would I say it? It's like electricity, I guess. An electrical, I don't know, an electrical shock type of pain. [Adult, Age 41]
Adult	1	1 (100%)	
Child	0	0 (0%)	
*M-CM/MCAP*			The achy pain that I get sometimes and, um, er, I'd say the, er, the overgrowth. Um, it's affected my feet so it's caused, um, me getting shoes to become difficult. Ah, usually in my legs. Um, sometimes it's the back of my legs, um, and it's not very often that it happens; it's just once in a while. [Adult, Age 25]
Adult	2	1 (50%)	
Child	3	0 (0%)	
*FAVA*			…so in my case, it is in my left glute muscle area, um, and it is in my upper thigh, um…in the back part of my thigh, so up by my hamstring, um…and I experience a lot of swelling, pain, um, so having pain, nerve pain, um, the nerve pain is very shooting, um, sometimes stabbing pain, um. The skin can get, I mean, mostly it's swelling. Um, I get some sciatic pain that shoots down my leg and I get, er, it impacts my mobility. [Adult, Age 25] But the pain goes up into my, erm, upper thigh, and to my left butt cheek… My calf, my ankle, my foot, erm. Yeah, it kind of just spreads all over, erm…definitely, painful, sore. Usually—not a day'll go by without pain. Constant pain, a lot of the time. There are very, very few times I'm not in any pain. [Child, Age 14]
Adult	1	1 (100%)	
Child	5	5 (100%)	
*LM*			Um, before I got the bump on my tongue cut off, it used to be very bad, like I couldn't eat because it would swell up so much, but now it's fine. [Child, Age 10]
Adult	0	0 (0%)	
Child	2	2 (100%)

#### PROMIS 29 + 2 profile v2.1

Five out of 10 (50%) adult participants found all domains of the PROMIS Profile to be relevant to the symptoms and impacts they experienced with PROS. Eight of out 10 (80%) said the anxiety and depression domains were relevant. Items related to average symptoms, which asked, “how run down were you on average, how fatigued were you on average,” were reported by a single participant as difficult to answer due to variability in symptoms throughout the day. However, most adult participants (n = 9; 90%) reported no difficulty with the items. Nine out of 14 (64%) children said all domains and items were relevant and had no difficulty comprehending or responding to items.

#### PROMIS sleep disturbance short form (pediatric)

Sleep items were not included in the pediatric version of the PROMIS Profile and therefore the PROMIS Sleep Disturbance form was debriefed in addition to the PROMIS Profile. Nine out of 14 (64%) children reported these items as relevant, and none had difficulty comprehending or responding to items.

#### PROMIS dyspnea severity

All adult participants found the dyspnea items to be easy to respond to and comprehend. Five out of 10 (50%) reported these items as relevant. Of those adults who reported the dyspnea items as not relevant, all had either KT or CLOVES. Of 11 children asked, 6 (55%) reported these items as relevant and of 12 asked, all reported no difficulty with the items as written.

### Age threshold for self-report and observer-report COAs

Twelve out of 20 (60%) child participants were able to self-report without caregiver help. Five children (25%) could not complete the COAs due to difficulties comprehending and responding to items. In these instances, the interviewer switched to the observer-report versions, though that switch occurred at different points for each interview. Those who were able to consistently self-report without difficulty were over the age of 12. While two 10-year-olds were able to complete all COAs via self-report, one required coaching and assistance from the caregiver. Three of the 20 (15%) child interviews (an 8-year-old and 14-year-old, both with M-CM, and a 6-year-old with MCAP) were completed entirely with the caregiver, due to cognitive, neurological, or developmental delays associated with the condition, that prevented the child from comprehending and responding to items. The majority of children over the age of 12 were consistently able to self-report and complete all COAs without difficulty. Caregivers who participated when their children were unable to self-report described the observer-report versions as relevant, easily understood, comprehensive, and appropriate for reporting observations of their child’s symptoms.

## Discussion

### Primary study objective

The overarching goal of this study was to determine the appropriateness and fit-for-use of the selected COAs to measure symptom severity and disease-related impacts in individuals with PROS. Self-report, pediatric, and observer-report versions of the COAs were tested. Given the multiple syndromes in which the PIK3CA mutation can be expressed, this study had the challenging task of testing whether a broad range of COAs and items appropriately and comprehensively captured the heterogeneity of symptoms experienced by persons with PROS.

A few items and domains were experienced less frequently by some participants: dyspnea, depression, and anxiety. Many others reported these domains as relevant, thus it is important to retain such items to capture the full range of experiences across syndromes. For example, shortness of breath has been identified as an important concept particularly for persons with lesions in the torso, despite 50% of the current sample reporting the dyspnea items as not relevant. Participants also reported that the type of pain they experienced was not captured in the current battery and would need to be assessed to gather an accurate picture of how they experience pain. Many participants preferred to identify multiple locations for pain using the BPI diagram, as their pain was not always limited to a single location. While only the paper version was tested in these interviews, the use of an electronic version of the BPI may offer a better opportunity for reporting pain in multiple locations. Based on the entire corpus of data collected, the full range of COA items comprehensively captured the range of symptoms and functional limitations experienced across PROS syndromes and severity levels. Overall, participants indicated items were relevant and demonstrated the ability to comprehend and respond to items.

Participants, across syndromes, described variability, both within and across days, for symptoms such as pain. For instance, one adult participant explained “my feelings are different in the morning than they are in the evening—you can average them, but the average doesn't make sense, because every morning, you know, I feel better than every afternoon, or every evening.” Such variability can impact responses and the ability to respond to items. Many preferred to report the worst pain they experienced rather than the average pain, as they were concerned that average pain would fail to reflect the severity of their symptoms. Participants also had mixed impressions of the PGI-S. Concerns were expressed about using a single state scale to rate overall severity, given the multiple symptoms experienced that vary in severity throughout the day. By including other COAs, with a variety of questions and recall periods, along with the PGI-S (as was the case with this battery), the severity of more specific symptoms (e.g., pain) was captured.

### Secondary study objective

A secondary objective of this study was to determine the appropriate age of self-report for participants. Those children who were able to consistently self-report, without difficulty, were over the age of 12. While two 10-year-olds were able to complete all COAs via self-report, one required coaching and assistance from the caregiver. Age based recommendations for PRO administration consider whether the PRO is developed for the target age group, but also how children’s developmental stage may impact their ability to comprehend and report on health data. Findings from this study are consistent with the ISPOR Task Force Report on Good Research Practices for the Assessment of Children and Adolescents which states that self-report is often inconsistent and unpredictable for 8–11 year-olds, due to developmental differences in cognitive capacity to respond to health questionnaires; self-report on health is reliable at the ages of 12 and older [23].

The protocol for this study assumed self-report for the adults, therefore, caregivers were not asked to participate with their adult children. However, participants with M-CM or MCAP had difficulty with self-report, irrespective of age, due to developmental delays and other neurological problems. All 14-year-olds diagnosed with syndromes other than M-CM or MCAP were able to self-report without difficulty; one 14-year-old diagnosed with M-CM was not able to self-report and required observer-reporting from the caregiver. During the screening process, some adults were also unable to independently self-report and therefore could not participate. Specifically, 3 caregivers of adults showed interest in having their adult child participate in the study but expressed concerns about their adult child’s ability to participate independently. For example, one 20-year-old was completely non-verbal due to neurological involvement. In these circumstances, observer-report COAs could conceivably be used in future studies to capture patient data from caregivers.

Observer-reported interviews were conducted during this study when it was determined that the child participant was unable to self-report. Those interviews support the use of observer-report COAs with caregivers and confirmed their ability to report on their child’s symptoms.

### Study limitations

Efforts were made to mitigate impacts of study limitations, though these limitations should be acknowledged. While a purposive sampling approach was used to recruit a mix of demographics, including race, ethnicity, and sex to ensure that the instruments being tested were understood and appropriate across various subgroups, participants were mostly female and Caucasian/White. Though existing literature does not indicate any prevalence patterns or differences in symptoms and impacts in PROS for race or sex [5, 24–26], we recognize that health outcomes, and related measurement tools, are subject to biases from health disparities. As this study tested existing, already-established measures that have been widely used and validated in other health conditions, with more diverse populations, we did not expect comprehension in this sample to differ from the general population or from those with other health conditions in which these measures were tested [15–21]. This study also only included English speaking individuals in the US. It cannot be assumed, therefore, that findings are representative of non-English speaking individuals and/or a non-US-based sample. The study sample was, however, diverse in terms of US geographic region (North, South, East, and West). The study was designed to oversample CLOVES and KT by including 40–60% of participants with either of these syndromes, as the literature recognizes CLOVES and KT as the most prevalent compared to other syndromes. While the overall sample included fewer participants in other syndromes, the aim of the study was not to saturate concepts within syndromes or to make comparisons between them, but instead to ensure representation to confirm appropriateness of the instruments across syndromes. Disease severity classifications were not identified for participants due to differences in how severity is determined for each syndrome. Interviews did, however, reveal a wide array of symptoms and impacts, including those who experienced moderate or severe limitations in everyday activities.

Other recruitment limitations should be noted. Participants’ diagnoses of PROS were self-reported rather than clinician-confirmed. While obtaining confirmation of diagnosis directly from physicians is ideal, it can be a lengthy and costly process, and can further challenge recruitment in rare conditions such as PROS. Recruitment through patient organizations and use of screening and interview questions specifically for persons with PROS helped maximize the likelihood that participants had a confirmed diagnosis. Recruitment through patient organizations can also impact the type of individuals who elect to participate. Those who actively engage with these organizations may experience more severe manifestations of the disease that prompted them to seek support and they could be more educated on the available treatments. This could influence their experience of the condition and the representativeness of this sample.

Three final limitations related to interview procedures should be recognized. Participants were sent the battery of COAs via email with their reminder 24-h prior to their scheduled interview. They were instructed not to review the instrument items prior to the interview. Though it is possible that some participants may have opened the document prior to their scheduled interviews, we do not expect prior knowledge of the items to bias results. Second, CD interviews are often conducted in person to capture nonverbal and behavioral nuances important for interpretation. Given the ambulatory challenges associated with PROS, combined with the goal of achieving diversity in symptom severity, phone or webcam interviews were conducted. To mitigate potential limitations of this strategy, interviewers were trained to recognize changes in tone, inflection, and lengthy pauses that could indicate challenges understanding and/or responding to an item. While technical familiarity prevented some from participating by webcam, no differences in participant responses across these two modes were noted. Lastly, a few child/caregiver interviews at the end of the study occurred during the COVID-19 pandemic. The experience of restricted movement outside of the home, including school, extracurricular activities, and socializing with peers, impacted some participants’ responses to the COAs during CD, though participants were aware that their responses to these items were influenced by the pandemic. Though a potential limitation, this awareness further demonstrated their ability to comprehend the intended meaning of the concepts being measured.

## Conclusion

Findings from this study provided valuable qualitative evidence from the perspective of persons with PROS about their experiences with symptoms such as pain and other disease-related impacts. Identifying COA measures with a broad enough range of concepts and items appropriate for measuring this set of rare diseases and its accompanying diversity of symptoms is a challenge. This was the only study, to date, to address this challenge and provide valuable insight on measures appropriate for capturing the similarities and diversities across these syndromes. Overall, the COAs tested (See Table 1) measured concepts that were relevant to PROS, using items that were easily understood. Evidence from these interviews supported the content validity of the tested COAs, with the recommended revisions and considerations for self-report thresholds for age and syndrome (See Table 4). The COAs tested in this study were appropriate and useful for measuring the symptoms and impacts of PROS in this heterogeneous patient population, in clinical trials and other research.

## Data Availability

The datasets generated and/or analyzed during the current study are not publicly available but are available from the corresponding author upon reasonable request.

## Abbreviations

BPI: Brief pain inventory
CD: Cognitive debriefing
CLOVES: Congenital lipomatous overgrowth, vascular malformations, epidermal nevi, scoliosis/skeletal and spinal
COA: Clinical outcome assessment
CLVM: Capillary, lymphatic, and venous malformations
FAVA: Fibro-adipose vascular anomaly
HRQoL: Health-related quality of life
ISPOR: International Society for Pharmacoeconomics and Outcomes Research
KT: Klippel-Trenaunay syndrome
LM: Lymphatic malformation
MCAP/M-CM: Megalencephaly-capillary malformation
PGI-S: Patient global impression of severity
PROMIS: Patient-reported outcomes measurement information system
PROS: PIK3CA related overgrowth spectrum
US: United States of America
